# NBI cystoscopy and bipolar electrosurgery in NMIBC management – An overview of daily practice 

**Published:** 2013-06-25

**Authors:** B Geavlete, F Stănescu, C Moldoveanu, M Jecu, L Adou, C Bulai, C Ene, P Geavlete

**Affiliations:** "Sf. Ioan" Clinical Emergency Hospital, Department of Urology

**Keywords:** “en bloc" resection, bipolar TURBT, plasma-button vaporization, NBI cystoscopy

## Abstract

Introduction: The standard non-muscle invasive bladder cancer (NMIBC) endoscopic diagnosis suffers from the frequently unsatisfactory white light evaluation accuracy leading to residual lesions being left behind. Monopolar transurethral resection of bladder tumors (TURBT) is marked by a substantial morbidity rate requiring further amelioration.

Methods: Small size tumors (under 1 cm) are feasible for “en bloc" resection. Bipolar TURBT was defined as being able to challenge the gold-standard status of monopolar resection due to the reduced complication rates. Plasma-button vaporization was introduced as a promising large bladder tumors’ ablation modality. Narrow band imaging (NBI) cystoscopy became an increasingly popular method of enhancing tumor detection.

Results: The “en bloc" resection of small size or thin pedicle tumors provides the conditions for avoiding tumoral tissue scattering. Bipolar resection is characterized by decreased perioperative bleeding risks and faster patient recovery. Plasma-button vaporization gained confirmation as an innovative approach, able to dispose large tumor bulks under complete control while minimizing the associated morbidity. NBI cystoscopy is a useful tool in identifying CIS lesions, small papillary tumors or extended margins of large tumor formations. As a cost-free technique, it may be extensively used both during the NMIBC initial diagnostic as well as during follow-up evaluation protocol.

Conclusions: Having in mind the various modalities of ameliorating the bladder cancer diagnostic and treatment, NMIBC management should be tailored in accordance with the particularities of each case.

## Introduction

Despite the technological advances achieved in the field of endourology, non-muscle invasive bladder cancer (NMIBC) continues to represent a challenging pathology, generally characterized by disappointing clinical outcomes and alarmingly high recurrence rates. The main reasons behind the relatively unsuccessful NMIBC therapeutic management are represented by the clearly doubtful oncological safety of the transurethral resection of bladder tumors (TURBT) [**1**] as well as by the insufficient accuracy of standard white light cystoscopy (WLC) [**2**]. 

 From this perspective, TURBT has been characterized as based on the “incise and scatter" oncologically flawed principle [**3**] and, despite numerous attempts of improvement, continued to constitute the standard NMIBC treatment alternative during the past 50 years [**4**]. Moreover, the bladder mucosa endoscopic inspection under simple white light was often described as a rather blunt tool in detecting urothelial malignant lesions, thus creating the premises for leaving behind the future “recurrences" [**5**].

 Based on these premises, the aim of the present paper was to analyze the potential means of improving the NMIBC diagnostic and therapy while considering both the bladder tumors’ standards of care eventually improved by observations from daily surgical practice as well as the most recent technological progresses.

### “En bloc" resection – Preferable when feasible

**Fig. 1:**
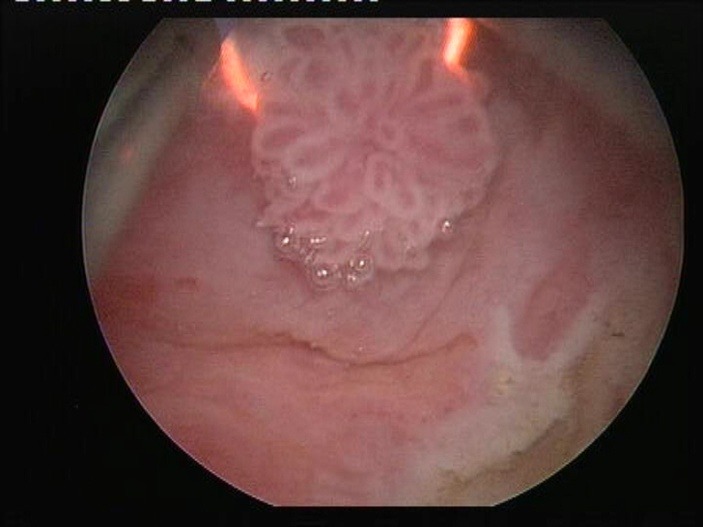
“En bloc" bipolar resection of a small size pTaG1 tumor

Tumor size obviously dictates the type of technical modality aimed to carry out the malignant tissue ablation process. In cases of small tumors less than 1 cm, “en bloc" removal by standard single wire resection loop is the most indicated approach, as it prevents tumoral cells from scattering by extracting the lesion in a single piece (**Fig. 1**). 

 In these situations, a special attention must be given to the complete and deep resection of the tumoral base while including part of the underlying muscular layer in the specimen.

 Although various endoscopic instruments have been reported as effective in such cases [**6**], our expertise relies mostly on the simple loop TUR, which we came to appreciate as both a safe and effective tool. This type of procedure may also be attempted in tumor formations between 1 and 3 cm in diameter and presenting a small implantation base (preferably a thin pedicle), while lesions over 3 cm or sessile tumors with a wide surface base do not usually have indication for this technique.

### Bipolar resection – Definitely a “must" in bladder cancer transurethral surgery

 Bipolar energy is most certainly recommended as the future of TURBT by the growing experience and increasingly numerous reports available in literature [**7**]. Our experience during the past 5 years confirmed these findings and imposed bipolar TUR as a standard of “good clinical practice" in NMIBC treatment.

**Fig. 2:**
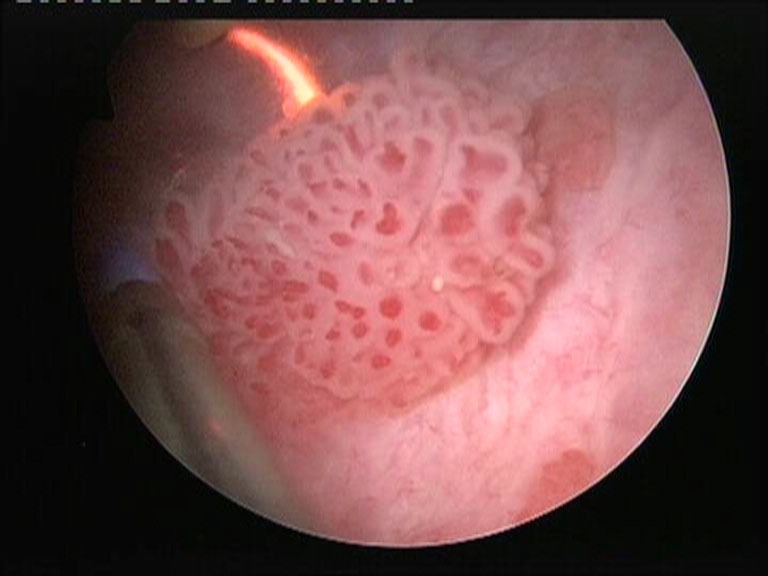
Bipolar TURBT of a lateral bladder wall lesion

 The main advantages of bipolar resection over the monopolar approach are well defined by the published trials: reduced perioperative bleeding risks, superior haemostatic abilities and faster postoperative recovery [**8**]. Subjectively speaking, we noticed that, during bipolar TURBT, a cleaner tissue cut is achieved, thus enabling a more accurate and precise tumor ablation to be performed. This feature is particularly useful during the resection of the thinned tumoral bed area, of tumors located on the lateral (**Fig. 2**) or posterior bladder walls implying a high perforation risk as well as in cases of extensive flat lesions widely spread out throughout the bladder mucosa surface.

 Bipolar TUR is indicated in virtually all NMIBC patients, while our practice focused especially on tumors smaller than 3 cm, thus leaving larger formations for a different approach (to be subsequently discussed). We routinely apply a staged resection beginning with the exofitic part of the tumor and continuing with the tumoral base and the separate sampling of biopsy specimens intended to certify complete tumor ablation.

### Bipolar plasma vaporization of bladder tumors – “A coat for the rainy days" in NMIBC management

 Endoscopic monopolar vaporization of tumors was at some point introduced as a viable NMIBC therapeutic alternative [**9**] but eventually failed to gain acknowledgement in clinical trials. During the past 5 years, the plasma-button vaporization became increasingly popular as an efficient benign prostatic hyperplasia (BPH) treatment and was eventually oriented towards large bladder tumors’ management as well [**10**].

 From the very beginning, we noticed that the bipolar plasma vaporization technique is able to provide the conditions for a fast, safe and efficient malignant tissue ablation phenomenon to be achieved. In our view, the BPV-BT cases selection criterion was represented by patients preoperatively diagnosed as presenting at least one apparently non-muscle invasive bladder tumor over 3 cm in diameter based on abdominal ultrasound, computer tomography and flexible WLC [**11**].

**Fig. 3:**
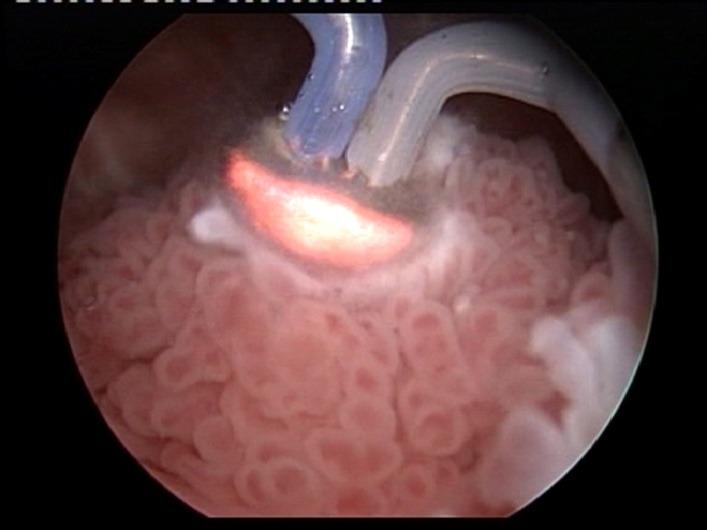
Bipolar plasma vaporization of the large tumor bulk – Initial aspect

 From the technical point of view, the plasma-button vaporization provides the conditions for the large tumor bulk to be gradually removed, layer by layer, starting from the very surface (**Fig. 3**) and progressing down to the tumor base (the “hovering" technique). 

**Fig. 4:**
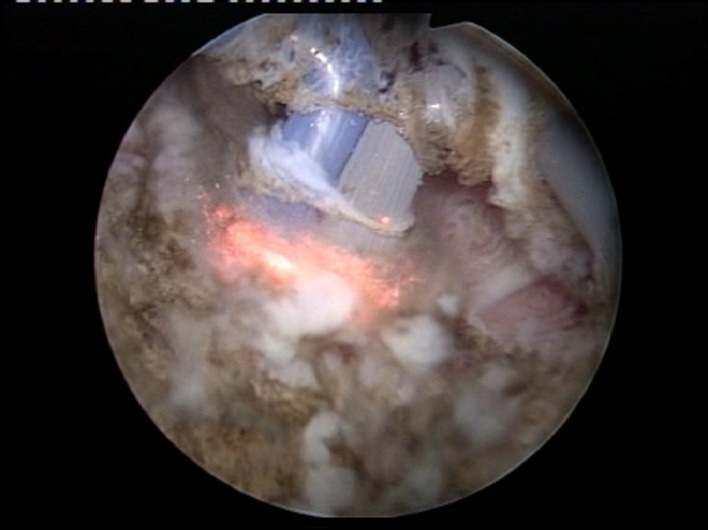
Advanced stages of the BPV-BT approach

 As to the actual upper hand over standard resection, the key is constituted by the remarkable endoscopic visibility maintained throughout the entire tumor ablation process. Practically, the tumoral tissue is melted down by the plasma corona displayed on the surface of the “button" electrode under complete visual control from the operator (**Fig. 4**).

**Fig. 5:**
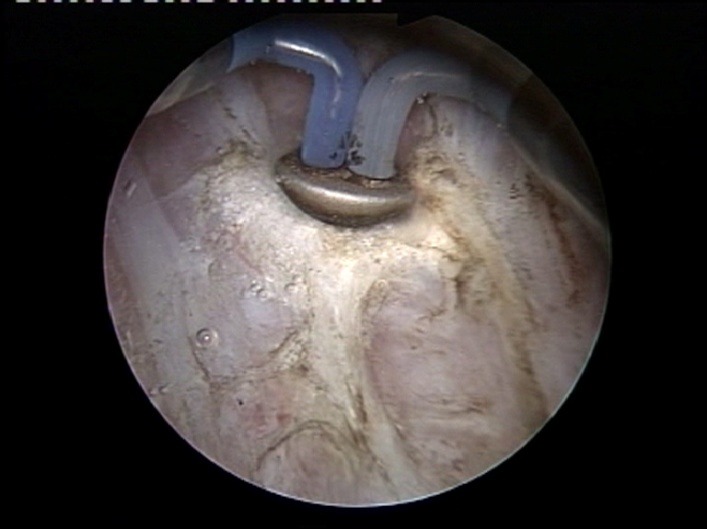
Plasma-button coagulation of the entire vaporization area

The coagulation of any hemorrhagic sources is basically concomitant and eventually followed by the larger vessels’ hemostasis (**Fig. 5**).

 The clinical research in this particular endourological field established the BPV-BT evidence based advantages: reduced obturator nerve reflex and related bladder wall perforation risk, decreased perioperative bleeding rate due to the superior coagulation properties of the plasma-button, reduced convalescence period and fewer short-term recurrences when compared to standard TURBT patients [**12**].

**Fig. 6:**
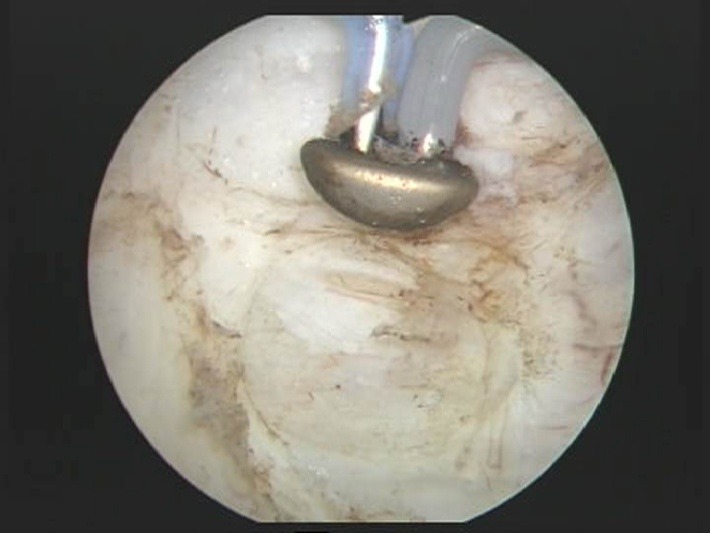
Clean muscular layer of the bladder wall after complete tumoral tissue removal

 One important issue that requires clarification is represented by the somewhat debatable oncological safety of a tumor vaporization technique. From the strictly surgical point of view, at the end of the BPV-BT procedure, as a rule, we emphasize the clean, tumor-free aspect of the muscular fibers of the bladder wall (**Fig. 6**). 

**Fig. 7:**
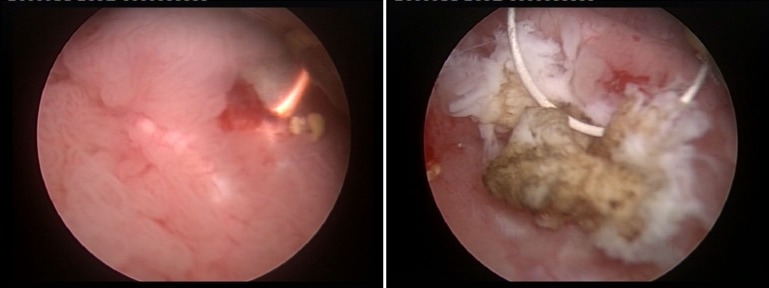
Deep biopsy resection of a large tumor including part of the underlying muscular layer

 From the perspective of the pathological analysis, before initiating the vaporization process, a deep biopsy resection of the tumor formation is performed and the obtained specimens are specifically intended to include part of the underlying muscular layer (**Fig. 7**). 

**Fig. 8:**
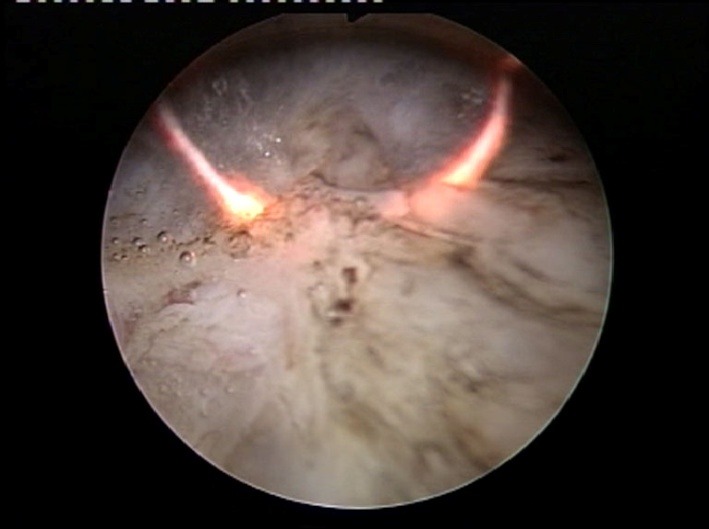
Tumoral bed area standard biopsy resection

 In this manner, the correct tumor stage is defined in each case with the utmost certainty, as shown during the course of two BPV-BT randomized trials completed so far [**11,12**]. Last, but not least, the complete malignant tissue ablation subsequent to plasma-button vaporization is confirmed during the last step of the technique in question, namely the bipolar biopsy resection from the center and margins of the vaporization area (**Fig. 8**).

### NBI cystoscopy – A cost-free, repeatable mean of improving tumor detection

 Narrow band imaging (NBI) cystoscopy was developed as a complementary method to the standard WLC, aimed to improve the bladder tumors’ detection solely based on an optic technological advancement [**13**]. The fact that it did not require the instillation of an expensive contrast agent (otherwise the main drawback of the photodynamic diagnostic – PDD) represented a substantial advantage from the very beginning.

In short, the NBI visualization mode is based on the white light being filtered into 2 discrete centre wavelengths for blue (415 nm) and green (540 nm), otherwise strongly absorbed by hemoglobin and only able to penetrate the tissue surface [**14**]. Therefore, the vascular structures appear dark brown (capillary vessels) and green (veins), thus contrasting with the pink or white background of normal mucosa [**15**]. Subjectively, NBI technology was shown to accurately underline the specific vascular architecture of urothelial lesions, thus creating the impression of a three-dimensional visualization of tumors and clearly defining their limits [**16**].

 Essentially, both according to literature [**17**] as well as to our own published data [**18**], NBI cystoscopy was proved to overcome the standard WLC diagnostic accuracy by finding more tumors. Patients with carcinoma in situ (CIS) lesions particularly benefited from the respective technique (**Fig. 8**), which displayed a substantially increased detection rate in comparison with the white light evaluation [**19**]. 

**Fig. 9:**
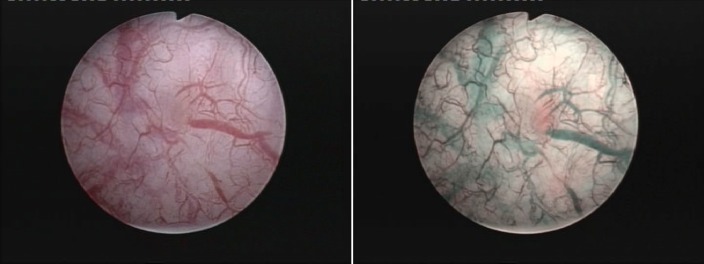
CIS lesion missed during the white light inspection and only found in NBI mode

 Otherwise, small pTa (**Fig. 9**) or even pT1 tumors may be missed during WLC and only discovered with the help of the NBI mode.

**Fig. 10:**
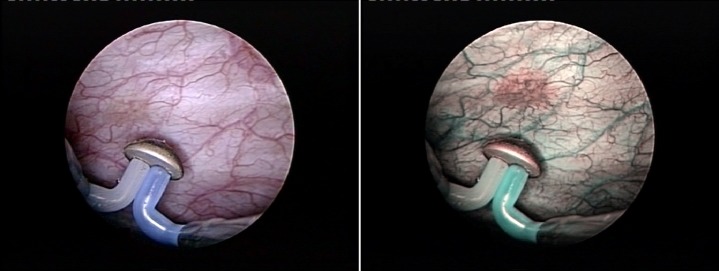
Follow-up NBI cystoscopy revealing a recurrent pTaG3 lesion missed during standard WLC

 Moreover, according to our protocols, a special indication for the NBI evaluation is represented by cases of large bladder tumors (over 3 cm), widely acknowledged as associating hardly visible extended tumoral margins (**Fig. 10**) as well as satellite or simply concomitant small lesions [**20**]. In such situations, NBI cystoscopy displayed superior detection rates over WLC in a randomized clinical setting, brought a significant contribution to an improved oncological outcome and reduced medium term recurrence rates being eventually obtained [**12**]. 

**Fig. 11:**
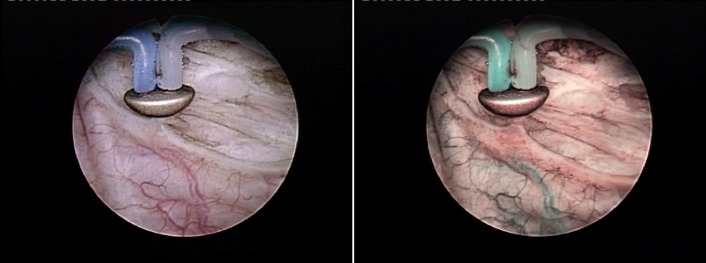
Extended positive tumoral margins only emphasized during the NBI control

 As far as the daily urological practice is concerned, the NBI visual assessment is indicated both during the initial diagnostic at first NMIBC suspicion as well as part of the standard follow-up evaluation protocol, regardless of tumor stage or grade. 

## Conclusions

In 2013, bladder cancer is still far from becoming a solved health problem for modern urology. The frequently poor NMIBC evolution and worryingly elevated tumor recurrence rates seriously question the gold-standard status of white light cystoscopy and monopolar TURBT. When feasible, “en bloc" resection could end the poor oncological safety profile of the “incise and scatter" principle behind TUR. Bipolar TURBT may represent at least a partial answer to the shortcomings of monopolar resection in terms of surgical safety and patient comfort. Plasma-button vaporization definitely shows some rather promising premises of ameliorating the large bladder tumors’ endoscopic management. As far as the NMIBC detection accuracy is concerned, NBI cystoscopy appears to outline a viable alternative of improving standard WLC results while concomitantly surpassing the PDD related heavy economic burden. Most importantly, it has become clear that the diagnostic and therapeutic NMIBC management should be tailored based on the particularities of each patient and adapted to its evolution.

 ACKNOWLEDGEMENT: This paper is supported by the Sectoral Operational Programme Human Resources Development (SOP HRD) 2007-2013, financed from the European Social Fund and by the Romanian Government under the contract number POSDRU/107/1.5/S/82839".
